# Flourine-18 Prostate-Specific Membrane Antigen-1007 Positron Emission Tomography Imaging in Staging of Primary and Secondary Prostate Cancer—A Retrospective Observational Cohort Study

**DOI:** 10.1097/JU9.0000000000000206

**Published:** 2024-10

**Authors:** Matthew H. V. Byrne, Nithesh Ranasinha, Abhishek Sharma, Claudia Mercader, Mutie Raslan, Ganesh Sathanapally, Francesca Lewis, Stefanos Gorgoraptis, Ana Catarina Lopes Vieira, Jedrzej Golebka, Bryony Peiris, J. Francisco Lopez, Tom Leslie, Richard C. Bell, Saiful Miah, Mark Tuthill, Andrew Protheroe, Philip Camilleri, Ami Sabharwal, Gerard Andrade, Freddie C. Hamdy, Ruth Macpherson, Fergus Gleeson, Richard J. Bryant, Alastair D. Lamb

**Affiliations:** 1 Oxford University Hospitals NHS Foundation Trust, United Kingdom; 2 Nuffield Department of Surgical Sciences, University of Oxford, United Kingdom

**Keywords:** PSMA-PET, fluorine-18, prostate cancer, staging

## Abstract

**Background:**

Detection of metastatic disease is important to inform prostate cancer management.

**Objectives:**

Evaluate local and distant staging by initial ^18^F-PSMA-1007 PET in primary and secondary prostate cancer.

**Design, Setting, and Participants:**

We retrospectively identified a consecutive series of ^18^F-PSMA-1007 PET scans from the date of introduction of ^18^F-PSMA-1007 PET in September 2019 until April 2022 at a single UK tertiary referral center. Our protocol was registered in advance (OSF registration ID: KTE3R).

**Results:**

We identified 1335 PSMA-PET scans, from 1220 men. Across 623 initial scans for primary staging, we observed PSMA-PET avidity in 97.6% cases positive for local disease, 29.5% for nodal disease, and 26.5% for metastatic disease. PSMA-PET identified a 13.2% absolute increase in nodal lesions compared with MRI and a 24.0% absolute increase in metastatic lesions compared with MRI marrow. The sensitivity for detection of local disease among 79 patients who had radical prostatectomy was 96.2% for PSMA-PET vs 89.4% for multiparametric MRI. Across 612 scans for secondary staging, we observed PSMA-PET positive avidity in 51.2% of cases for local recurrence, 46.6% for nodal disease, and 43.0% for metastatic disease. When evaluated by the PSA range for patients receiving secondary staging, using the PSA values of 0.2 to 0.49, 0.5 to 0.99, 1 to 1.99, and ≥ 2 ng/mL, PSMA-PET scans were positive in 57.8%, 75.0%, 83.8%, and 95.5% of cases, respectively. PSMA-PET identified a 26.2% absolute increase in metastatic lesions compared with MRI marrow or other skeletal MRI (n = 61) and a 14.7% absolute increase in metastatic lesions compared with the bone scan (n = 42).

**Conclusion:**

^18^F-PSMA-1007 PET identifies a higher number of nodal and metastatic lesions compared with conventional cross-sectional imaging. However, the high number of indeterminate lesions and stage migration necessitates discussion of ^18^F-PSMA-1007 PET imaging within a multidisciplinary team and places a higher burden on these teams.

PROSTATE cancer (PCa) is a leading cause of cancerrelated disease burden among men worldwide.^1^ It is a heterogenous malignancy, requiring careful grading and staging to identify and treat clinically significant disease while avoiding overtreatment of nonclinically significant disease. The European Association of Urology guidelines recommend the use of abdominopelvic imaging and bone scintigraphy to assess metastatic disease in higher risk PCa.^2^ However, multiparametric MRI (mpMRI) and CT have low sensitivity,^3,4^ and MRI has low specificity for detecting lymph node metastasis.^5–7^ This is problematic as patients considered appropriate for radical therapy for localized disease may have micrometastases.^8^ Positron emission tomography (PET) can detect local and distant PCa foci due to increased expression of prostate-specific membrane antigen (PSMA) on the surface of PCa cells.^9^ Most PSMA-PET imaging uses gallium-68 (^68^Ga) ligand-bound PSMA.

PSMA-PET is established in the setting of secondary PCa staging. A systematic review of 37 studies (comprising 4790 patients) by Perera et al^10^ demonstrated that in patients with biochemical recurrence (BCR), ^68^Ga-PSMA-PET positivity increased with increasing PSA level and could be used to detect recurrence at formally defined BCR PSA levels (^68^Ga-PSMA-PET positivity was evident in 75% of those in the PSA range 1-1.99 ng/mL, compared with only 33% for <0.2 ng/mL). There is also evidence to consider replacing conventional imaging modalities with PSMA-PET for primary PCa staging.^11–13^ For example, the ProPSMA trial, a randomized controlled trial of 302 men with high-risk primary PCa, evaluated the accuracy of ^68^Ga-PSMA-PET vs CT and bone scintigraphy in the detection of pelvic nodal or distant metastatic disease. ^68^Ga-PSMA-PET had significantly superior accuracy (92% vs 65%, *P* < .0001), as well as higher sensitivity (85% vs 38%) and specificity (98% vs 91%), compared with CT and bone scintigraphy.^12^

A more recent development in PSMA-PET imaging is the use of fluorine-18 (^18^F) as an alternative tracer to ^68^Ga, with ^18^F having a longer half-life, thus permitting longer-distance transport to the site of use, and a higher image resolution.^14^ It has also reduced renal clearance, potentially improving detection of disease near the urinary tract.^15^ However, a limitation of ^18^F-PSMA-1007 is that there is higher potential for uptake in benign tissue^16^ (Table 1), and the false positive rate can vary with the specific ^18^F ligand used.^13^

The evidence base for ^18^F-PSMA-1007 PET is growing. Small studies have investigated its utility for locoregional and distant staging in both primary and secondary PCa, comparing it with conventional imaging and histopathology, and stratifying by the PSA level.^18–22^ Larger studies have focused on ^18^F-PSMA-1007 PET in secondary prostate cancer.^23^ Saule et al^24^ reviewed ^18^F-PSMA-1007 PET for secondary prostate cancer and demonstrated a detection rate of 80% to 87.5% in 7 studies ranging in size from 21 to 251 patients. Mingels et al^25^ evaluated ^18^F-PSMA-1007 PET in 177 patients with BCR and identified a positive rate of 91% for local, nodal, or metastatic disease and sensitivity of 95% (95% confidence interval [CI] = 90%-98%) and specificity of 89% (95% CI = 83%-93%) compared with histology; PSA decrease after treatment; or persistent lesion on follow-up imaging. Notably, Mingels et al demonstrated a sensitivity of 97% (95% CI = 85%-99%) but a specificity of only 74% (95% CI = 57%-85%) for bone metastases.^24,25^ As such, ^18^F-PSMA-1007 may identify small lesions but may have nonspecific uptake in bone, for example, in ribs. Giesel et al^26^ investigated ^18^F-PSMA-1007 PET in 251 patients with BCR after prostatectomy. Similar to the findings of Perera et al with the ^68^Gallium ligand, detection rates with ^18^F-PSMA-1007 increased with the PSA level, from 61.5% at 0.2 to 0.49 ng/mL to 94.0% at ≥ 2 ng/mL. Giesel et al^26^ identified local recurrence in 19.5%, pelvic lymph node involvement in 40.6%, and bone metastasis in 40.2% of patients.

Our center has performed ^18^F-PSMA-1007 PET since 2019, and we have conducted all completion primary staging for patients with Gleason grade group ≥ 3, stage ≥ T3a, and PSA ≥ 20 ng/mL since August 2020. An illustrative image of our pathway is shown in Figure S1 (http://links.lww.com/JU9/A78). In this study, we aimed to examine the added value of ^18^F-PSMA-1007 PET over conventional staging modalities for local, nodal, and distant PCa in primary and secondary staging.

## Patients and Methods

We retrospectively identified a consecutive series of ^18^F-PSMA-1007 PET scans. All ^18^F-PSMA-1007 PET scans were performed for PCa staging at a single UK tertiary referral center, from the date of its introduction in September 2019 until April 2022. There was a minimum of 6-month follow-up to identify subsequent histology and corroborating scans. The protocol was registered in advance (OSF registration ID: KTE3R)^27^ and followed the Strengthening the Reporting of Observational Studies in Epidemiology guidelines for cohort studies (Appendix A, http://links.lww.com/JU9/A80).^28^

Data were extracted from electronic patient records using a proforma (Appendix B, http://links.lww.com/JU9/A81). For primary staging, we compared PSMA-PET with mpMRI and RP histology (for T and N staging) as the “ground truth,” and MRI marrow (for M staging). For secondary staging, we compared PSMA-PET with MRI marrow and other skeletal MRI (eg, MRI spine) or bone scintigraphy (for M staging). A review of PSMA-PET was limited to the first PSMA-PET for each patient for primary and secondary prostate cancer. A review of other imaging modalities was limited to the previous 3 years.

In our center, we use whole-body MRI marrow to identify skeletal metastases instead of bone scintigraphy or CT of the abdomen and pelvis for patients with a PSA ≥ 20. We use this modality as it is more accurate than bone scintigraphy and CT for detecting skeletal metastases.^29^

High PSMA-PET avidity has been reported in benign prostates.^16^ We sought to mitigate this by stratifying scans as positive, indeterminate, or negative for PCa based on information provided by the radiologist in their report. PSMA-PET was used for completion staging in primary disease with any of the following higher-risk disease features (in patients with a performance status 0-1): Gleason grade group ≥ 3, stage ≥ T3a, PSA ≥ 20 ng/mL, and absence of widespread metastases on prior MRI/CT. We used PSMA-PET in secondary disease staging in the context of BCR (PSA > 0.2 ng/mL) post RP, Phoenix criteria postradical radiotherapy (ie, a PSA rise > 2 ng/mL above the nadir), or based on clinical suspicion of disease recurrence.

Narrow slice MRI scans focusing on indeterminate regions of interest identified on the PSMA-PET were introduced later in our series (starting May 2021). These consisted of MRI as T1 and STIR sequences through the region of interest in a range of 4 to 5 mm slice thicknesses. The narrow slice MRI scans were performed when there was diagnostic uncertainty from the PSMA-PET scan images and were used as a “ground truth” in this study.

### Ethics

This study was approved as a service evaluation by the Audit Department at Oxford University Hospitals NHS Foundation Trust, UK. Ethical approval was not required for this study.

### Analysis

Descriptive analyses presented as number and percentage were calculated using R (version 4.1.0),^30^ and specificity and sensitivity analyses were performed in MedCalc (version 20.112).^31^ For primary PCa, a T stage of 2 was considered positive for all imaging modalities and we have performed a separate analysis for PIRADS ≥ 3 disease for mpMRI.

## Results

We identified 1335 ^18^F-PSMA-1007 PET scans performed for 1220 men. Of these, 623 were the first PSMA-PET scans for primary staging and 612 were the first PSMA-PET scan for secondary staging. We excluded 100 PSMA-PET scans that were performed after the initial PSMA-PET scan for primary and secondary staging.

### Primary Staging

The mean age of patients with primary PCa was 70.0 years (SD = 7.4 years), and median PSA at time of diagnosis = 13.0 ng/mL (IQR = 7.3-27.9 ng/mL). NCCN risk classification was possible for 552 men; 29.2% were intermediate-risk and 70.8% were high-risk.

Of the 623 PSMA-PET scans for primary PCa, 97.6% were positive for local disease, 29.5% for nodal disease, and 26.5% for metastatic disease. We then stratified positive avidity by NCCN risk stratification. Among patients with intermediate-risk disease, 7.5% (12 of 161 men) had nodal disease and 14.9% (24 of 161 men) had metastatic disease on PSMA-PET. Among patients with high-risk disease, 37.9% (148 of 391 men) had nodal disease and 29.7% (116 of 391 men) had metastatic disease on PSMA-PET.

**Figure 1:**
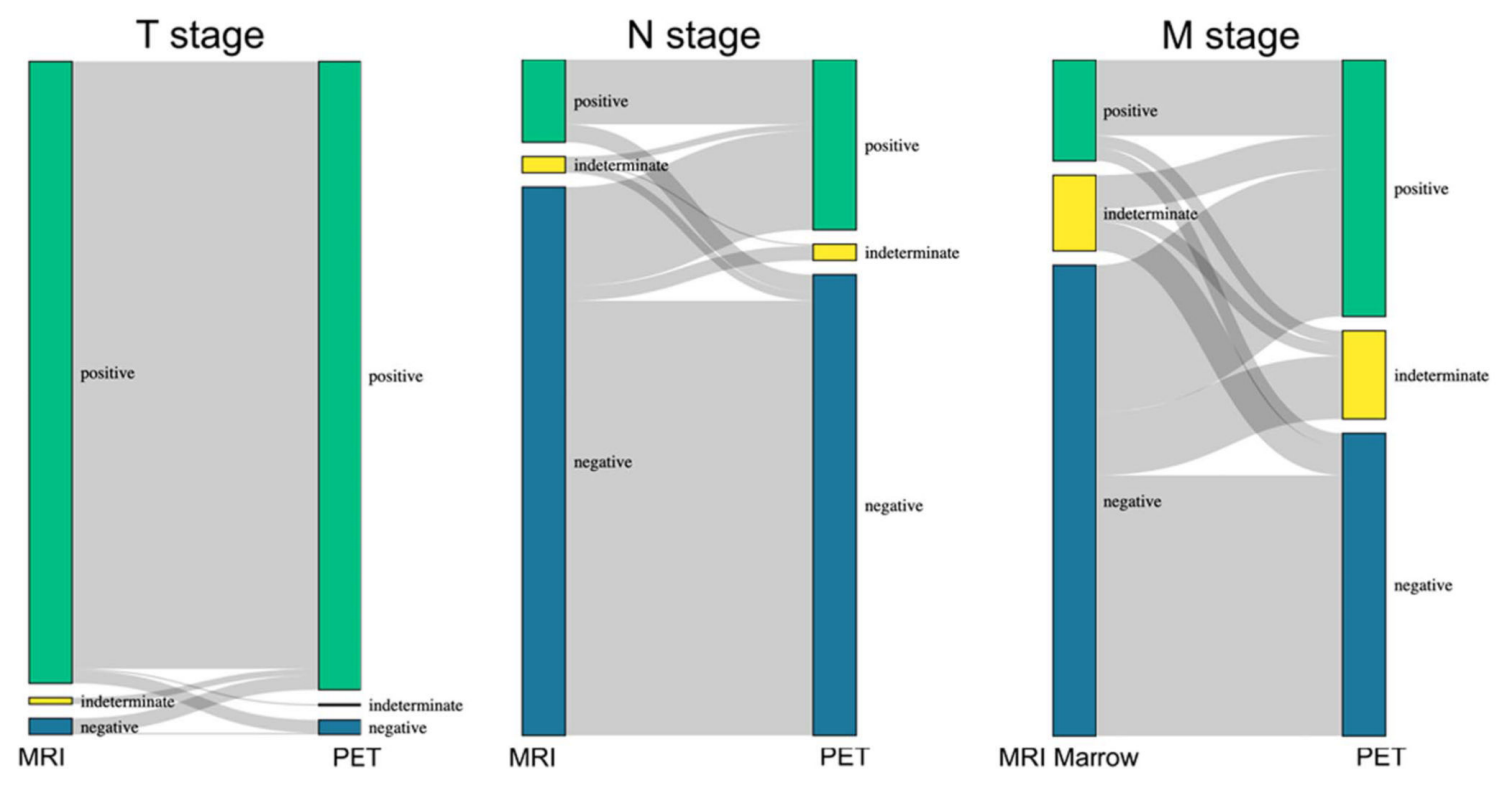
Sankey diagram demonstrating difference between primary prostate cancer staging with ^18^F-PSMA-1007 PET vs MRI (n = 410) for T and N staging, and vs MRI marrow for M staging (n = 154). PSMA, prostate-specific membrane antigen; PET, positron emission tomography.

In 410 patients who had both PSMA-PET and mpMRI performed, PSMA-PET identified local disease similar to mpMRI (95.1% vs 94.2%), respectively, for all patients with T stage ≥ 2 on mpMRI. PIRADS data were available for 332 of the 410 patients. For patients with PIRADS ≥ 3 on mpMRI, detection was 97.0% for PSMA-PET and identified a 13.2% absolute increase in nodal lesions compared with mpMRI (25.6% vs 12.4%, respectively). A total of 154 patients had both PSMA-PET and MRI marrow. PSMA-PET identified a 24.0% absolute increase in metastatic lesions compared with MRI marrow (39.6% vs 15.6%, respectively) (Figure 1).

The location of the metastatic bone lesions varied between pelvic bones, ribs, and other isolated areas, as well as multisite skeletal metastases (Table 2).

Seventy-nine men underwent RP, and all cases had clinically significant prostate cancer (grade group 2 or above). Fifty-nine of 66 men had PIRADS ≥ 3 disease on mpMRI (missing PIRADS data for 13 men referred from outside the region or no recent mpMRI scan data available). Seventy-six of 79 men had positive PSMA-PET scans for local disease (no missing data), giving a sensitivity of 89.4% (95% CI = 79.4%-95.6%) for mpMRI and a sensitivity of 96.2% (95% CI = 89.3%-99.2%) for PSMA-PET for detecting local disease. We were unable to calculate specificities as there were no true negatives.

Six-week post-RP PSA values were available for all 79 men who had RP. Four patients had a 6-week PSA ≥ 0.1 ng/mL, all of whom had a positive surgical margin. Four patients had a preoperative PSMA-PET scan positive for metastatic disease (but negative conventional imaging), and in these 4 patients, the PSA at 6-week post-RP was ≤ 0.01, suggesting that these may have been false positive imaging results.

Narrow slice MRI was used to corroborate skeletal lesions in 18 patients with PSMA-PET scans. Only 2 narrow slice MRI scans were positive, highlighting the issue of false positivity for PSMA-PET scans.

We selected men who had grade group ≥ 3 disease on their biopsy and PSA < 20 and > 20 ng/mL (Table 3). We then compared N stage and M stage for PSMA-PET vs mpMRI and MRI marrow, respectively. For N stage for patients with PSA < 20 ng/mL, PSMA-PET identified nodal disease in 36 of 211 patients and mpMRI identified nodal disease in 21 of 211 patients. For M stage for patients with PSA < 20 ng/mL, PSMA-PET identified metastatic disease in 16 of 40 patients and MRI marrow identified metastatic disease in 7 of 40 patients. We identified all primary PSMA PET imaging who also had an MRI marrow, of the 151 patients, 50 had M1b or M1c on PSMA PET and 19 were positive on MRI marrow. We then stratified by PSA and were not able to identify a threshold where that differentiated positive from negative imaging. As such it does not appear as though we can omit ^18^F PSMA PET based on a PSA threshold (Table S1, http://links.lww.com/JU9/A79).

A surprising finding from our cohort was that 7 of 25 patients with a PSA > 100 had no evidence of metastatic disease on PSMA-PET or MRI marrow (Table S2, http://links.lww.com/JU9/A79). This was re-reviewed by our multidisciplinary team (MDT) radiologist for the purposes of validation of data, who drew the same conclusions as MDT staging. It will be interesting to report on the disease outcomes of this group in the future.

### Secondary Prostate Cancer

The mean age of patients with secondary PCa was 71.0 years (SD = 7.4), and median PSA at time of PSMA-PET was 1.9 ng/mL (IQR = 0.4-6.5 ng/mL). The principal treatment was RP in 58.1%, radiotherapy in 28.5%, brachytherapy in 4.4%, and chemotherapy, hormonal, or other treatments in 9.0% of 597 men.

PSMA-PET was positive in 51.2% of cases for local recurrence, 46.6% for nodal disease, and 43.0% for metastatic disease. When evaluated by the PSA range, using the PSA values of 0.2 to 0.49, 0.5 to 0.99, 1 to 1.99, and ≥ 2 ng/mL, PSMA-PET scans were positive in 57.8%, 75.0%, 83.8%, and 95.5%, respectively (Table 4).

**Figure 2:**
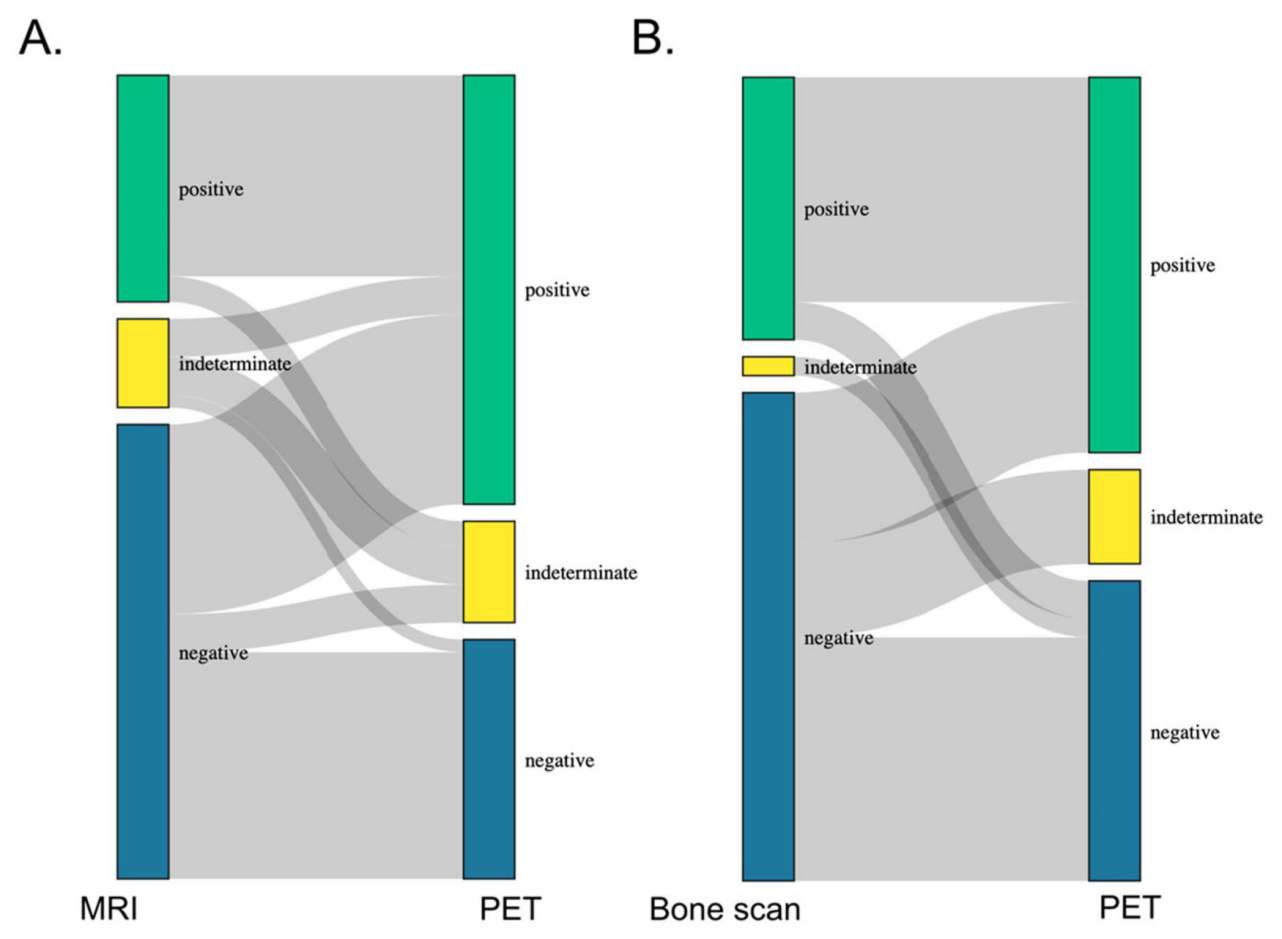
Sankey diagram demonstrating difference between secondary prostate cancer staging with ^18^F-PSMA-1007 PET vs **(A)** MRI marrow or other skeletal MRI (n = 61) for M staging or **(B)** bone scan for M staging (n = 42). PSMA, prostate-specific membrane antigen; PET, positron emission tomography.

In men who had PSMA-PET and MRI marrow or other skeletal MRI (n = 61), or bone scintigraphy (n = 42), PSMA-PET identified a 26.2% absolute increase in metastatic lesions compared with MRI marrow or other skeletal MRI (55.7% vs 29.5%, respectively) and a 14.7% absolute increase in metastatic lesions compared with bone scintigraphy (48.8% vs 34.1%, respectively) (Figure 2).

## Discussion

To our knowledge, our study comprises the largest cohort investigating the clinical utility of ^18^F-PSMA-1007 PET to date.

### Primary Disease

In primary PCa, this study demonstrates that ^18^F-PSMA-1007 PET is comparable with mpMRI in identification of local disease (95.1% vs 94.2% positive for PCa, respectively), and when compared with the histology of 79 RP samples, ^18^F-PSMA-1007 PET had a higher sensitivity (96.2% vs 89.4%, respectively). Our data support the existing literature on ^68^Ga-PSMA-PET for primary local PCa staging, which shows it adds value over mpMRI in the identification of nodal and metastatic disease as well as local clinically significant PCa, albeit with an increase in false positives.^13^

Our data set demonstrated that ^18^F-PSMA-1007 PET identified a greater number of nodal lesions than MRI and a greater number of metastatic lesions than MRI marrow in patients with primary PCa. In a study of 96 men with PCa undergoing RP with lymphadenectomy or salvage lymphadenectomy, ^18^F-PSMA-1007 PET had a high specificity (>99%) for nodal metastasis.^18^ Similar findings were reported in a prospective comparison between ^18^F-PSMA-1007 PET and whole-body MRI in the nodal staging of 79 men with intermediate-risk or high-risk PCa; ^18^F-PSMA-1007 PET had a significantly higher sensitivity in identifying nodal metastasis than MRI, with a sensitivity of 87% (95% CI = 71%-95%) with ^18^F-PSMA-1007 PET compared with 37% (95% CI = 22%-55%) with MRI.^32^ In our study, when patients were stratified by NICE/NCCN risk stratification, several ^18^F-PSMA-1007 PET scans were positive in patients with intermediate-risk disease, suggesting that ^18^F-PSMA-1007 PET should not be solely reserved for high-risk patients. We have not formally reported our lymphadenectomy outcomes because of conservative local practice toward lymphadenectomy and conservative surgical practice for node-positive prostate cancer. However, 5 of 31 patients who had lymphadenectomy had node-positive disease, and PSMA-PET was positive in 1 of 5 of these cases, with none of the 31 patients having node-positive disease on mpMRI.

^18^F-PSMA-1007 PET is prone to demonstrating avidity in nonclinically significant PCa or benign tissues.^16^ Although we aimed to mitigate this through reporting positive, indeterminate, and negative lesions based on information provided by the radiologist in their report rather than any avidity, we found a large number of indeterminate lesions. Consequently, we introduced narrow slice MRI later in our series, and 2 of 18 narrow slice scans were positive for metastatic disease. Moreover, our 6-week post-RP PSA data showed 4 of 79 patients were false positives for metastatic disease. This highlights the need for discussion of all ^18^F-PSMA-1007 PET imaging within a multidisciplinary team that includes experienced radiologists who are familiar with ^18^F-PSMA-1007 PET imaging, and the added value of a radiologist with dual expertise in both PSMA-PET and mpMRI.

### Secondary Prostate Cancer

In secondary PCa, we demonstrate that ^18^F-PSMA-1007 PET identified a greater number of metastatic lesions than conventional imaging. This is consistent with a previous study demonstrating that ^18^F-PSMA-1007 PET outperforms bone scintigraphy in identifying metastatic disease (100.0% vs 50.0%, respectively).^21^ Our data provide further evidence of the efficacy of ^18^F-PSMA-1007 PET in the identification of PCa recurrence in patients with BCR.^20^

When the detection rate of ^18^F-PSMA-1007 PET for any disease was stratified by the PSA range, we observed that overall the ^18^F-PSMA-1007 ligand performed similarly to the ^68^Ga ligand in the data reported by Perera et al and a study of ^18^F-PSMA-1007 PET in 251 patients with BCR after RP by Giesel et al (Table 4).^10,26^ In the lower PSA ranges (0.2-0.49, 0.5-0.99, and 1.0-1.99 ng/mL), our data suggest that ^18^F-PSMA-1007 PET has a higher detection rate than ^68^Ga-PSMA-PET when compared with previous data.^10^ This is consistent with the findings of other recent studies. In men with BCR after RP and a PSA level < 0.5 ng/mL, the detection rate was 57.9% for ^68^Ga-PSMA-PET,^33^ while a detection rate of 86% was reported for ^18^F-PSMA-1007 PET in men with BCR within the same PSA range.^20^ However, it is important to caveat this point by mentioning the higher avidity rate associated with the ^18^F ligand.^16^

Data comparing both the ^68^Ga and ^18^F ligand suggest similar utility,^34^ but a higher rate of equivocal lesions with ^18^F-PSMA-1007. Alberts et al^35^ demonstrated a nonsignificant higher ^18^F-PSMA-1007 positivity rate than 68-Gallium (91.8% vs 86.9%, respectively, *P* = .68), but a significantly greater rate of “uncertain findings” (17.2% vs 8.3%, respectively, *P* = .02) in 244 patients with BCR. Pattison et al^36^ performed a similar study of 50 patients (initial staging, n = 12; BCR, n = 27; meta-static disease, n = 11) who had both ^18^F-PSMA-1007 and ^68^Ga PSMA-PET and demonstrated similar staging in 92% of patients but a higher number of equivocal results for ^18^F-PSMA-1007 than ^68^Ga (n = 7 vsn = 1, respectively). A study of ^18^F-PSMA-1007 PET identified an approximately 5-fold greater number of lesions of a benign origin than ^68^Ga-PSMA-PET in 102 patients receiving both imaging modalities following BCR after RP. ^18^F-PSMA-1007 PET identified 369 avid lesions, 245 of which were attributed to benign disease, whereas ^68^Ga-PSMA-PET identified 178 avid lesions, 52 of which were attributed to benign disease.^16^ These lesions were most frequently ganglia, nonspecific lymph nodes, and bone lesions.^16^

### Strengths and Limitations

This study has several strengths. First, the range of different imaging modalities against which ^18^F-PSMA-1007 PET was compared; second, the use of a predefined protocol; and third, the large size of the consecutive cohort data following introduction of ^18^F-PSMA-1007 PET at our center. The study also has several limitations. First, it is a pragmatic data set, with some missing data, particularly for patients referred for PSMA-PET imaging from other centers. This limits the reliability of comparisons between imaging modalities. Second, our center does not routinely use Likert scoring for PSMA-PET. Indeterminate lesions were considered negative lesions, an approach that has been employed elsewhere for meta-analysis.^11^ This may result in an underestimate of the number of false positives. Our selection criteria include absence of widespread metastases on prior MRI or CT, which may increase the percentage difference between the 2 imaging modalities. Finally, our study lacks a definitive “ground truth” in the nodal and metastatic setting, as defined by gold-standard histopathological diagnosis (of metastatic lesions), to corroborate true positives or negatives for our cases—which is rare outside a clinical trial. The lack of a definitive ground truth is a common limitation for studies in this area. Rather, we used corroborating narrow slice MRI as our ground truth. We were not able to evaluate whether there was an institutional learning curve or trend in the accuracy of PSMA positive scans correlating with positive pathology as prostatectomy samples were positive and the number of nodal samples was too low.

For some patients, we identify disease stage migration (Figures 1 and 2), but we cannot know for certain how this affects clinical outcomes without long-term studies of ^18^F-PSMA-1007 PET performed alongside a standard-of-care imaging cohort, or without metastatic biopsies. We appear to have changed one group of indeterminate patients on conventional imaging to another group of indeterminate patients on ^18^F-PSMA-1007 PET. This can add more uncertainty for the MDT. In the United Kingdom, most centers are subject to limitations in the availability of radioisotopes, leading to delays in staging and treatment for patients with prostate cancer. Whether this translates into clinical benefit remains to be seen. Therefore, there is a need to rationalize the use of PSMA-PET, perhaps using MRI marrow for individuals with a high PSA because sensitivity of conventional imaging is high in this setting, whereas we might reserve PSMA-PET for patients who have a low PSA or a negative initial MRI marrow despite a high PSA. Identification of early nodal and metastatic disease is challenging, and it is important to offer patients the most appropriate treatment modality. ^18^F-PSMA-1007 PET provides another tool for multidisciplinary teams to use to inform their treatment decisions, and consequently, we believe it can benefit patients overall. However, from our experience, we have found a higher burden is placed on the multidisciplinary teams to distinguish clinically significant disease from false positives and nonsignificant avidity on PET scans—as gold standard histopathology of metastatic lesions is rare outside a clinical trial. As gallium-68 and some other fluorine-18 ligands have a lower false positive rate than ^18^F-PSMA-1007 PET,^16^ centers should consider their MDT capacity and expertise alongside other advantages and disadvantages when deciding on their choice of ligand for PSMA-PET (Table 1).

## Conclusion

In primary PCa staging, ^18^F-PSMA-1007 PET adds value over mpMRI in identification of nodal and distant metastatic spread as well as local disease. In secondary PCa staging, ^18^F-PSMA-1007 PET can identify the site of recurrence or progression after primary treatment at similar rates to ^68^Ga-PSMA-PET. However, due to the high number of indeterminate lesions and stage migration compared with standard imaging, a higher burden of responsibility is placed on multidisciplinary teams to distinguish clinically significant disease from indeterminate or benign imaging features in ^18^F-PSMA-1007 PET images. This necessitates the need for discussion of ^18^F-PSMA-1007 PET imaging within a multidisciplinary team that includes experienced radiologists who are familiar with ^18^F-PSMA-1007 PET imaging and the added value of a radiologist with dual expertise in both PSMA-PET and mpMRI.

## Patient Summary

In this study, we evaluated how a scan performed at detecting prostate cancer in the prostate and detecting prostate cancer that had spread to other parts of the body. We found that the scan we used detected more areas that could be cancer than other types of scans, but it was not always clear whether those areas had cancer. This means the scan we used needs to be discussed in a meeting with a group of experts to decide whether these areas had cancer.

## Take Home Messages

^18^F-PSMA-1007 PET identifies a high number of indeterminate lesions and stage migration compared with conventional cross-sectional imaging.

This places a higher burden of responsibility on multidisciplinary teams to distinguish clinically significant disease.

## Supplementary Material

SDC1

SDC2

SDC3

SDC4

## Figures and Tables

**Table 1:** Comparison of Different PET Tracers for Prostate Cancer Imaging.^17^

PET modality	Mechanism of targeting		Mechanism of clearance	Sites of physiological uptake	Advantages	Disadvantages
	Tracer	Target				
^68^G-PSMA-11 PET	Gallium-68 radioisotope T½ 68 min	PSMA	Renal	Kidney, bladder, salivary/lacrimal glands, liver, spleen, intestines	High prostatic specificity	Higher urinary clearance
^18^F-PSMA- 1007 PET	Fluorine-18 radioisotope T½ 110 min	PSMA	Hepatic	Kidney, salivary/lacrimal glands, liver, spleen, intestines	High prostatic specificity; minimal urinary clearance; longer half-life; higher image resolution	Potential for uptake and PET avidity in benign/clinically insignificant tissue
^18^F-DCFPyl PET	Fluorine-18 radioisotope T½ 110 min	PSMA	Renal	Similar to ^68^G-PSMA-11 but with lower renal uptake	Lower skeletal uptake	Higher urinary clearance

DCFPyl, 2-3-{1-carboxy-5-[(6-18F-fluoro-pyridine-3-carbonyl)-amino]-pentyl}-ureido)-pentanedioic acid; PSMA, prostate-specific membrane antigen; PET, positron emission tomography.

**Table 2:** Location of Suspected Skeletal Metastasis on ^18^F-PSMA-1007 PET. The Total Number of Suspected Lesions is Provided, as Well as Those Who Were Positive and Indeterminate on ^18^F-PSMA-1007 PET Imaging. Percentages are Given as a Percentage of the Total, With Location Data Available for 201 Men.

Location of suspected skeletal metastasis on ^18^F-PSMA-1007 PET	Total, n	Positive			Indeterminate	
		n	%		n	%
Multiple other	120	99	82.5		21	17.5
Isolated pelvic or sacral	36	24	66.7		12	33.3
Isolated rib	21	4	19.0		17	81.0
Isolated spine	12	6	50.0		6	50.0
Isolated femur	8	5	62.5		3	37.5
Isolated sternum	2	2	—		0	—
Isolated scapula	1	0	—		1	—
Isolated humerus	1	0	—		1	—

PSMA, prostate-specific membrane antigen; PET, positron emission tomography.

**Table 3:** N and M Stage for Primary Prostate Cancer Patients With Grade Group ≥ 3 on Their Biopsy Stratified by PSA Level and Imaging Modality PSA (ng/mL) N stage

PSA (ng/mL)	^18^F-PSMA-1007 PET			mpMRI	
	n	%		n	%
N stage					
<20 (n = 211)	36	17.1		21	10.0
≥20 (n = 94)	49	52.1		19	20.2
	^18^F-PSMA-1007 PET			MRI marrow	
PSA (ng/mL)	n	%		n	%
M stage					
<20 (n = 40)	16	40.0		7	17.5
≥20 (n = 75)	31	41.3		10	13.3

PSMA, prostate-specific membrane antigen; PET, positron emission tomography; mpMRI, multiparametric MRI.

**Table 4:** Positive ^18^F-PSMA-1007 PET Imaging in Secondary Prostate Cancer for LR, Nodal, and Metastatic, and Any Disease. Perera et al used a ^68^Gallium Ligand and Giesel et al used a ^18^F-PSMA-1007 Ligand

Index treatment	PSA value (ng/mL)	LR (%)	Nodal (%)	Metastatic (%)	Any disease (n =)^a `^	Any disease (%)^a^	Perera et al (^68^Ga) Any disease (%)^10^	Giesel et al^26 ^(^18^F-PSMA-1007) Any disease (%)
Any	0.2-0.49	33.0	29.9	22.4	85 of 147	57.8	45	NA
	0.5-0.99	24.3	50.0	35.4	36 of 48	75.0	59	NA
	1-1.99	44.2	53.2	45.2	52 of 62	83.8	75	NA
	≥2	66.4	56.4	56.1	252 of 264	95.5	95	NA
RT and brachytherapy only	0.2-2	65.2	19.2	26.9	20 of 26	76.9	NK	NA
	≥2	73.3	47.2	55.6	137 of 144	95.1	NK	NA
RP only	0.2-0.49	28.6	32.4	21.3	76 of 136	55.9	46	62
	0.5-0.99	15.6	53.5	39.5	31 of 43	72.1	57	75
	1-1.99	27.3	61.9	45.2	36 of 42	85.7	82	91
	≥2	37.7	71.6	52.7	71 of 74	95.9	97	94

PSMA, prostate-specific membrane antigen; PET, positron emission tomography; LR, local recurrence; NA, not applicable; NK, not known; RP, radical prostatectomy; RT, radiotherapy.

a 91 excluded because of missing data, ie, PSA value at time of PET.

## Data

The data for this study has been deposited in a Mendeley Data repository, DOI: 10.17632/t46kfft7hd.1.
