# Supplementary figures and images for: Flourine-18 Prostate-Specific Membrane Antigen-1007 Positron Emission Tomography Imaging in Staging of Primary and Secondary Prostate Cancer—A Retrospective Observational Cohort Study
Source: JU Open Plus. Author manuscript; Available in PMC 2026 Mar 10. (PMC7618851; doi:10.1097/JU9.0000000000000206)

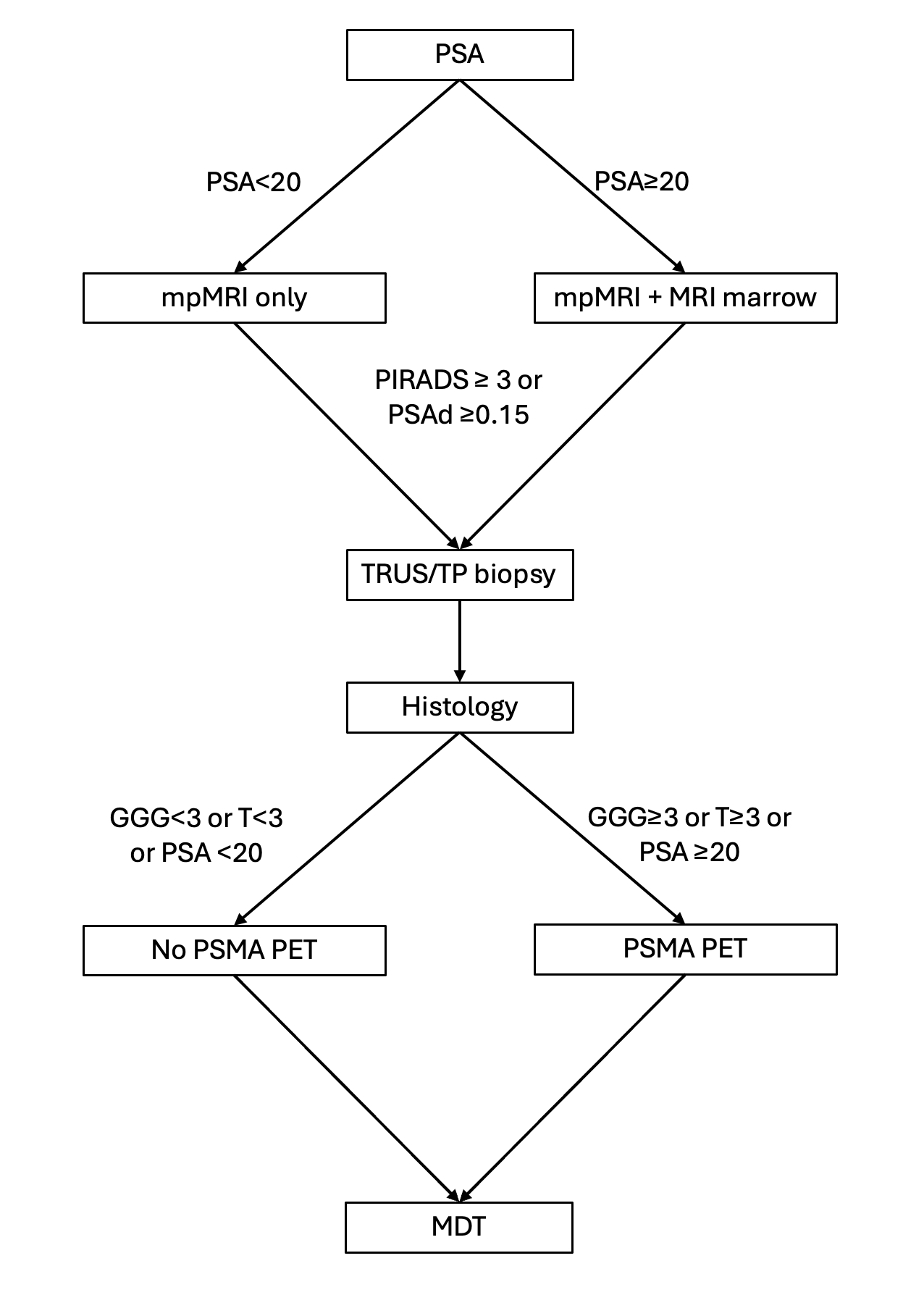


Figure S1: 18F-PSMA-1007 PET pathway at our centre

Supplement: SDC1 [file EMS212747-supplement-SDC1.docx]
