# Supplementary material for: Flourine-18 Prostate-Specific Membrane Antigen-1007 Positron Emission Tomography Imaging in Staging of Primary and Secondary Prostate Cancer—A Retrospective Observational Cohort Study
Source: JU Open Plus. Author manuscript; Available in PMC 2026 Mar 10. (PMC7618851; doi:10.1097/JU9.0000000000000206)
Supplement: SDC2 [file EMS212747-supplement-SDC2.docx]

Table S1: Primary PSMA PET and MRI Marrow positive for M1b+ disease stratified by PSA level. We identified all primary PSMA PET imaging who also had an MRI marrow, of them 50 had M1b or M1c on PSMA PET, and 19 were positive on MRI Marrow. We then stratified by PSA and were not able to identify a threshold where that differentiated positive from negative imaging.

| *PSA level* | *Total (n)* | *M1b or M1c PET (n)* | % | M1b PSMA PET (n) | % | *M1b Marrow*  *(n)* | % |
| --- | --- | --- | --- | --- | --- | --- | --- |
| *All PSA* | 151 | 50 | 33.1 | 47 | 31.1 | 19 | 12.6 |
| *PSA < 20* | 53 | 20 | 37.7 | 18 | 34.0 | 9 | 17 |
| *PSA >20* | 98 | 30 | 30.6 | 29 | 29.6 | 10 | 10.2 |
| *PSA 20 - 50* | 57 | 17 | 29.8 | 16 | 28.1 | 5 | 8.77 |
| *PSA >50* | 41 | 13 | 31.7 | 13 | 31.7 | 5 | 12.2 |

Table S2: Primary PSMA PET and MRI Marrow positive for N1 or M1+ disease stratified by PSA level. Across a total of 542 scans there was a general trend that the higher PSA, patients were more likely to have node positive or metastatic disease. Some patients had a PSA over 100, and approximately 30% were negative, these results were re-reviewed and confirmed with a Consultant Uro-radiologist that they were all negative on PSMA PET. The range of positive scans was smaller with MRI Marrow when stratified by PSA value (PSMA PET=27.9-72.0% versus MRI Marrow=17.5%-42.9%).

| *PSA level* | *Total PSMA PET (n)* | *N1 or M1+ PSMA PET (n)* | *%* | *Total MRI Marrow*  *(n)* | *N1 or M1+ MRI Marrow*  *(n)* | *%* |
| --- | --- | --- | --- | --- | --- | --- |
| *<10* | 222 | 62 | 27.9 | 32 | 7 | 21.9 |
| *10 - < 20* | 119 | 39 | 32.8 | 21 | 4 | 19.0 |
| *20 - < 50* | 122 | 61 | 50.0 | 57 | 10 | 17.5 |
| *50 - < 100* | 54 | 33 | 61.1 | 27 | 7 | 25.9 |
| *100 +* | 25 | 18 | 72.0 | 14 | 6 | 42.9 |
